# Shelf-Life Performance of Fish Feed Supplemented with Bioactive Extracts from Fermented Olive Mill and Winery By-Products

**DOI:** 10.3390/foods12020305

**Published:** 2023-01-09

**Authors:** Diogo Filipe, Margarida Gonçalves, Helena Fernandes, Aires Oliva-Teles, Helena Peres, Isabel Belo, José Manuel Salgado

**Affiliations:** 1 Centre of Biological Engineering, University of Minho, Campus de Gualtar, 4710-057 Braga, Portugal; 2 Faculty of Sciences, University of Oporto (FCUP), 4169-007 Porto, Portugal; 3 Interdisciplinary Centre of Marine and Environmental Research (CIIMAR), 4450-208 Matosinhos, Portugal; 4 Biotecnia Group, Campus Agua, Chemical Engineering Department, University of Vigo, Campus As Lagoas s/n, 32004 Ourense, Spain

**Keywords:** antioxidants, enzymes, functional aquafeeds, shelf-life, solid-state fermentation

## Abstract

Fortifying fish feeds with bioactive compounds, such as enzymes and antioxidants, has been an adopted strategy to improve feed nutritional quality and sustainability. However, feed additives can lose activity/effectiveness during pelleting and storage processes. This work aimed to monitor functional activity stability in feeds supplemented with a bioactive extract, including cellulases, xylanases, and antioxidants. This bioactive extract (FBE) was produced by *Aspergillus ibericus* under solid-state fermentation of olive mill and winery by-products. Two isoproteic and isolipidic diets were formulated and unsupplemented or supplemented with lyophilized FBE (0.26% *w*/*w*). Both diets were stored at room temperature (RT) or 4 °C for 4 months. Results showed that feed storage at 4 °C enhanced the stability of the enzymes and cellulase was more stable than xylanase. Compared to RT, storage at 4 °C increased cellulase and xylanase half-life by circa 60 and 14%. Dietary FBE supplementation increased antioxidant activity and storage at 4 °C reduced antioxidant activity loss, while in the unsupplemented diet, antioxidant activity decreased to the same level in both storage temperatures. Dietary supplementation with FBE reduced lipid peroxidation by 17 and 19.5% when stored at 4 °C or RT, respectively. The present study is a step toward improving the storage conditions of diets formulated with bioactive compounds.

## 1. Introduction

Fisheries and aquaculture support global fish production, both being key sectors to ensure food supply. Fisheries production reached a maximum and stagnated since the 1990s, while aquaculture has been on a steady rise [1]. This growth of aquaculture production has been raising concerns due to the reliance on fish meal and fish oil to produce aquafeeds [1]. These ingredients were mainly produced from whole fish. However, driven by the increased demand and price of these ingredients, as well as fish stock depletion, more fish by-products are used for fish meal and oil production, accounting for about 54% of European fish meal production [2]. Nowadays, plant feedstuffs have become the main ingredients in feeds, while fishmeal and oil have become high-value feedstuffs with strategic applications [3]. The main limitation of using plant feedstuffs in aquafeed is the presence of anti-nutritional factors such as non-starch polysaccharides (NSP) [4,5]. NSP may impair nutrient availability by increasing digesta viscosity [5], reducing access of digestive enzymes to the substrate, and slowing down gastrointestinal transit [5]. Furthermore, dietary NSP may also compromise fish growth performance and induce undesirable gut morphology and physiology alterations, impairing nutrient absorption and gut health [6].

Functional feed additives became a critical tool to surpass some of these challenges in settling sustainable aquaculture practices. Feed additives, including enzymes, organic acids, yeast products, probiotics, phytogenics, and mycotoxin binders, have a large array of compositions and applications [7]. Some feed additives may be used to increase the feed’s nutritional properties, reduce the anti-nutritional factors’ harmful effects, and promote feed consumption by the fish [8,9,10,11]. Others raise the possibility of including higher levels of more sustainable and cheaper ingredients, such as plant by-products [12]. And others may also enhance the fish’s immune and health status [13,14,15,16,17,18], thus reducing the need for antibiotics use in aquaculture [7].

The main functional feed additives used to increase NSP digestibility are enzymes, such as xylanases and cellulases. Some of these enzymes are authorized to be used as feed additives for chickens, turkeys, laying hens, minor poultry species, weaned piglets, pigs, and fish [19,20]. Positive effects of dietary xylanase and cellulase supplementation were observed in several fish species, for instance, by enhancing growth, feed digestibility, and digestive enzyme activities in yellow river carp (*Cyprinus carpio*) [21]; modulating gut microbiota of African catfish (*Clarias gariepinus*) [22]; increasing the digestibility of dietary dry matter, energy, total carbohydrates, and NSP in Nile tilapia (*Oreochromis niloticus*) [23]; and increasing digestibility of dry matter, energy, and starch in European Seabass (*Dicentrarchus labrax*), meagre (*Argyrosomus regius*) [24], white seabream (*Diplodus sargus*) [25], and turbot (*Scophthalmus maximus*) [26].

The main challenges of using enzymes as feed additives are their cost and stability during feed manufacturing and storage, which may decrease their efficacity due to denaturation processes [27]. One strategy to reduce the costs is the production of enzymes by solid-state fermentation (SSF) using low-cost agro-industrial by-products [28]. For example, cellulase production costs using SSF are circa 2.6-fold lower than traditional production by submerged fermentation [29]. SSF is a green bioprocess that occurs in the absence or near absence of free-running/circulating water [30]. The microorganisms better adapted to SSF are filamentous fungi due to their hyphal way of growth, allowing them to penetrate and disrupt the substrate and to have a high tolerance for low water activity conditions [31]. Fungi produce different types of exoenzymes, including lignocellulolytic enzymes like cellulase, xylanase, and β-glucosidases [32].

Quality management, from ingredient production to feed manufacture and processing, is essential for consistent and high-quality feed production. In addition, it is also crucial to ensure that the feed nutritional profile remains unchanged during storage [33]. However, many processes can negatively affect the quality of the feeds. Oxidation is one of these processes that may diminish feed quality during production and storage. Oxidation can occur by various mechanisms, such as autoxidation, photosensitized, thermal, and enzymatic oxidation. Feed contact with atmospheric oxygen reacts with radicals and causes autoxidation [34,35]. The ingestion of an oxidated lipids diet may induce several fish pathologies, such as liver degeneration, skeletal anomalies, anemia, hemolysis, and jaundice [36,37]. Long shelf storage of fish feeds, both at room and low temperatures may cause lipid degradation and decrease protein content [38]. Fish diets are rich in polyunsaturated fatty acids (PUFA) and are highly susceptible to lipid peroxidation [34,35]. Antioxidants have been added to diets to prevent lipid oxidation, increasing the shelf life of fish feeds [39,40]. Moreover, dietary antioxidants may also reduce reactive oxygen species during fish metabolism, avoiding deterioration of fish health status. Ethoxyquin (EQ) has been used in fish feed as an antioxidant [41]. However, the European Union recently banned its use due to concerns about the mutagenic and carcinogenic properties of this product [42,43]. This has led to the search for other antioxidant compounds capable of limiting the oxidation of lipids.

Wineries and olive mills in Europe account for most of the world’s olive oil and wine production [44,45], generating large amounts of by-products. The use of these by-products is currently limited due to their phytotoxic effects if used as fertilizers [46], and therefore they are typically burned as fuel. These by-products are rich in antioxidant compounds [47] bound to the plant cell walls [48,49], which reduces their bioavailability. SSF of these by-products with fungi may promote the release of cell wall-bound antioxidants and the production of enzymes, such as xylanase and cellulase [32,50]. From a circular economy strategy, the reutilization of these by-products is crucial for the environmental sustainability of these agricultural industries.

The evaluation of the stability of enzymes and antioxidants produced by SSF is critical to determine its dietary inclusion level, feed storage conditions, and shelf life. The stability of enzymes such as cellulase and xylanase is affected by feed manufacture, mainly associated with pelleting temperatures and storage conditions of feed. [51]. Adding the enzymes before feed pelleting promotes the homogeneous distribution of enzymes and their incorporation within the pellets. However, as aforementioned, the processing of pelleted diets can affect enzyme stability [52].

This work aimed to study the stability of a bioactive extract rich in cellulase, xylanase, and phenolic compounds obtained from the solid-state fermentation of winery and olive mills by-products. For that purpose, the extract was incorporated into a typical marine fish feed, and the cellulase, xylanase, antioxidant activity, and lipid peroxidation were evaluated during 183 days of storage at room temperature and 4 °C.

## 2. Materials and Methods

### 2.1. Raw Material and Microorganisms

A previously optimized mixture for producing bioactive compounds [53], including 30% exhausted grape marc, 36% vine shoot trimmings, and 34% exhausted olive pomace, was used as substrate in SSF with *Aspergillus ibericus* MUM 03.49.

### 2.2. Production of Bioactive Extract

The moisture of the substrate mixture was adjusted to 75% (*w*/*w*) and autoclaved (121 °C, 15 min) before SSF. Afterwards, 400 g of the mixture was placed in trays (dimensions 43 × 33 × 7 cm) and inoculated with 2 × 10^6^ spores of *Aspergillus ibericus* per gram of substrate mixture. SSF was carried out for 7 days at 25 °C. At the end of SSF, an aqueous extraction of the fermented substrate was performed (1 mL distilled water: 5 g of fermented mixture *w*/*v*) with constant stirring for 30 min. The mixture was then filtered through a fine-mesh net and centrifuged (11,200× *g*, 10 min, 4 °C). The supernatant was filtered by vacuum through filter paper (11 µm pore size), lyophilized (−51 ± 1 °C; 30–50 mTorr), and then stored at −20 °C until utilization. The lyophilized extract was denominated as FBE.

### 2.3. Experimental Diets

Two isoproteic (48% crude protein) and isolipidic (16% crude lipids) experimental diets were formulated, one unsupplemented (control diet) and another supplemented with 0.26% (*w*/*w*) FBE to contain 1550 U cellulase kg^−1^. Dietary ingredients were finely ground, mixed, and dry pelleted in a laboratory pellet mill (California Pellet Mill, Crawfordsville, IN, USA) with a 2 mm die. Pellets were then dried at 40 °C for 48 h to reduce the water content to circa 10%. The diets were stored at room temperature (averaging 25 °C) or at 4 °C in the dark. Two samples were taken from diets stored at both temperatures on day 1 and after 14, 28, 42, 56, 91, and 119 days of storage. The antioxidant activity, lipid peroxidation, and enzyme activity of xylanase, cellulase, and protease were screened at both temperatures in all sampling times.

Ingredient and proximate analyses of experimental diets are present in Table 1.

### 2.4. Xylanase and Cellulase Activity

Xylanase and cellulase activities were determined according to Fernandes et al. [8]. For xylanase, beechwood xylan (1% in citrate buffer, 0.05 N, pH 4.8) was used as the substrate, and carboxymethylcellulose (CMC) (2% in citrate buffer, 0.05 N, pH 4.8) was used as the substrate for cellulase. The reaction was performed at 50 °C for 15 min or 30 min for xylanase and cellulase, respectively, and free sugars were determined by the DNS method [54]. Calibration curves were made using concentrations between 0 g and 2 g of glucose or xylose L^−1^ for cellulase and xylanase, respectively, both in citrate buffer. In both procedures, one unit of activity was defined as the quantity of enzyme necessary to release 1 µmol of glucose/xylose per minute from the substrate at the reaction conditions. Xylanase and cellulase activity was expressed as units per gram of diet (U g^−1^).

### 2.5. Proteases Activity

Protease activity was determined using azocasein (0.5% *w*/*v* in sodium acetate buffer, 50 mM, pH 5) as substrate. Diet extract (0.5 mL) was mixed with 0.5 mL of azocasein and incubated at 37 °C for 40 min. After, the reaction was stopped by adding 1 mL of 10% trichloroacetic acid and centrifuged (1000× g, 15 min). The supernatants were recovered and mixed with 1 mL KOH 5 N, and the absorbance was read at 428 nm. One unit of protease activity was established as the amount of enzyme that increased by 0.01 the absorbance relative to the blank per minute under the assay conditions. Protease activity was expressed as units per gram of diet (U g^−1^).

### 2.6. Antioxidant Activity

Antioxidant activity was determined in vitro using the 2,2-diphenyl-1-picrylhydrazyl (DPPH) radical scavenging assay as described in [55]. The calibration was carried out with a concentration ranging from 100 µM to 2.125 µM of 6-hydroxy-2,5,7,8-tetramethylchroman-2 carboxylic acid (Trolox) mL^−1^. Results were expressed in millimoles of Trolox equivalents per kilogram of diet (mmol Trolox kg^−1^).

### 2.7. Lipid Peroxidation

Lipid peroxidation (LPO) was assessed using the method described by Nguyen [56]. Diets were homogenized with a thiobarbituric acid solution and placed at 95 °C for 15 min in a boiling water bath. Afterwards, the mixtures were cooled in ice-cold water, centrifuged (1500× g, 15 min, at 4 °C), and absorbance was read at 532 nm. Results were reported as mg malondialdehyde per kilogram of diet (mg MDA kg^−1^ diet).

### 2.8. Inactivation Parameters of Cellulases and Xylanases during Storage

The constant of the deactivation rate (*k_d_*) was analyzed following the first-order model described by Pal et al. [57] according to the following equation:

(1) $$\ln\frac{A}{A_{0}}=k_{d}\cdot \;t,$$

where *A*_0_ is the initial activity, *A* is the activity at each time, *k_d_* is the constant of deactivation rate (days^−1^), and *t* is the time of storage.

The half-life of cellulase and xylanase were calculated according to Equation (2):

(2) $$t_{1/2}=\frac{\ln2}{k_{d}},$$

where *t*_1/2_ is the half-life (days^−1^) and *k_d_* is the constant of deactivation rate (days^−1^). 

The decimal reduction time (*D-value*) was determined according to Equation (3):

(3) $$D-value=\frac{\ln10}{k_{d}}$$

### 2.9. Statistical Analysis

Data were checked for normality (Shapiro-Wilk test) and homogeneity of variances (Levene’s test) and normalized if necessary. The enzymatic and antioxidant activity were analyzed by one-way analysis of variance (ANOVA) and Dunnett’s test to discriminate significant differences against the initial storage time (*p* < 0.05). Dietary lipid peroxidation at each storage temperature was analyzed by two-way ANOVA, with diet and time as fixed factors. As the interaction was significant, the diet and time effects were analyzed by one-way ANOVA at each time and diet, respectively. For each diet and temperature, Dunnett’s test was used to discriminate significant differences in lipid peroxidation against the initial storage time (*p* < 0.05).

Data were analyzed with Statgraphics Centurion XVI software package for Windows and IBM SPSS statistics software package version 28.

## 3. Results and Discussion

### 3.1. Enzymatic Activities

After diet manufacturing (time 0 h), the xylanase and cellulase activities of the diet supplemented with FBE reached 2607 U kg^−1^ and 1080 U kg^−1^ diet, respectively.

**Figure 1:**
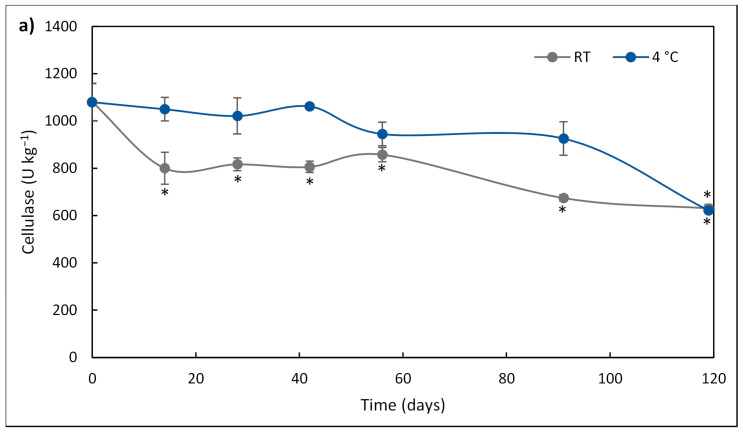

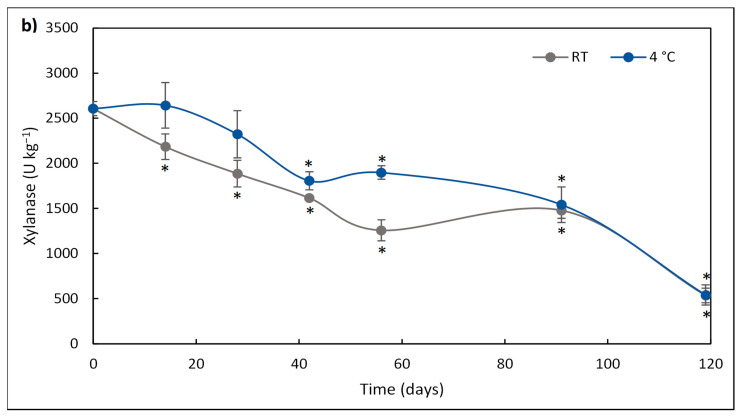
Cellulase (**a**) activity of FBE-supplemented diet over storage time (days) at room temperature (RT; square) and 4 °C (circles). The results represent the average of three independent experiments, and the error bars represent SD. The asterisk denotes a significant difference (*p* < 0.05) from the initial enzyme activity. Xylanase (**b**) activity of FBE-supplemented diet over storage time (days) at room temperature (RT; square) and 4 °C (circles). The results represent the average of three independent experiments, and the error bars represent SD. The asterisk denotes a significant difference (*p* < 0.05) from the initial enzyme activity.

Compared to the activity post-pelleting (time 0 h), the cellulase activity of the FBE-supplemented diet significantly decreased (*p* < 0.05) after 14 days of storage at RT, while at 4 °C it was stable for 119 days (Figure 1a). After 91 days of storage, the cellulase activity of the FBE-supplemented diet stored at 4 °C was 1.4-fold higher than that stored at RT.

The deactivation of xylanase activity of the FBE-supplemented diet, stored at RT and 4 °C, is presented in Figure 1b. The xylanase activity of the diet stored at 4 °C only decreased after 41 days, while that stored at RT decreased after 14 days. After 41 days, the loss of xylanase activity was higher at RT than at 4 °C. Xylanase activity of the diet stored at 4 °C was 1.5-fold higher than that stored at RT after 56 days of storage.

After 119 days of storage, enzymatic activity was still detected. Cellulase lost 41.7% and 42.4% of its initial post-pelleting activity at RT and 4 °C, respectively, while xylanase lost 79.5% and 79.2% of its initial post-pelleting activity at RT and 4 °C, respectively.

**Figure 2:**
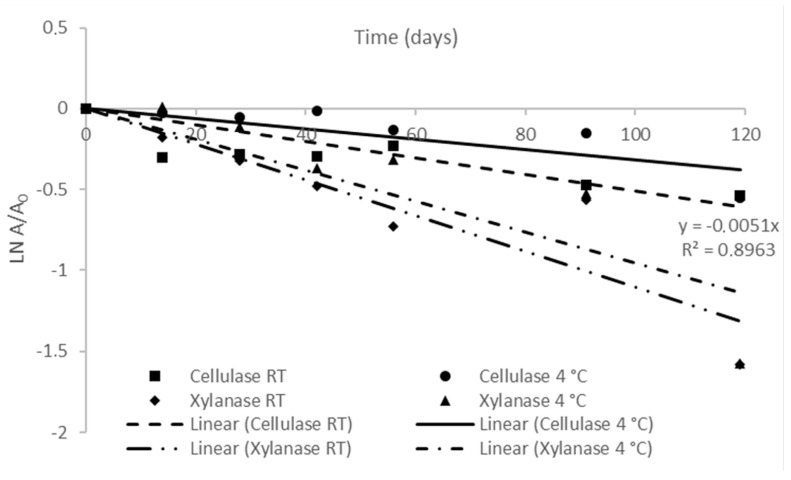
First-order storage deactivation of xylanase and cellulase activity of FBE-supplemented diet at room temperature and 4 °C. The results represent the average of three independent experiments and the error bars represent SD.

The kinetic parameters of xylanase and cellulase activity of the FBE-supplemented diet at RT and 4 °C are presented in Figure 2 and Table 2. For both enzymes, the deactivation over storage time followed a linear model (where R^2^ > 0.8133 in all enzymes and conditions). The deactivation rates of cellulase and xylanase (*k_d_*) were higher in the diet stored at RT than that stored at 4 °C, thus, the storage at 4 °C delayed the decrease of enzymatic activity. Half-life time (*t*_1/2_), representing the time required for the enzyme activity to be reduced to half of its initial value, of xylanase stored at 4 °C and RT was lower (9 days) than that of cellulase (81 days). Thus, refrigeration temperature had a higher impact on the increase in the *t*_1/2_ of cellulase than on the xylanase activity. The higher *t*_1/2_ of cellulase over xylanase showed that the stability of cellulase was higher than xylanase during storage, irrespective of temperature. Previously, studying the thermostability of enzymes production from SSF of brewer’s spent grain, it was observed that cellulase was more thermostable than xylanase when exposed to temperatures from 45 °C to 60 °C [58].

The first-order model also estimated the D-values, representing the time required for the enzyme activity to be reduced by 90% of its initial value. The higher D-value was achieved for cellulase activity of the diet stored at 4 °C, which was 1.6-fold higher than that stored at RT. Currently, dietary supplementation with exogenous enzymes has been increasing to improve the nutritional bioavailability of plant-based diets [59]. However, studies focusing on the stability of dietary enzymes during storage are still scarce. Fuente et al. [60] studied the storage of a barley-based diet for poultry supplemented with cellulases over 32 weeks at RT and observed a progressive reduction of enzyme activity, decreasing to 42% and 33% residual activity after 3 and 6 weeks of storage, respectively. In the present study, cellulase activity was reduced by 50% only after 19 weeks at RT and after 31 weeks at 4 °C.

Studies of the shelf-life of enzymes without their incorporation into a diet are more widely investigated. The higher stability of cellulase over xylanase was also observed by El-Sherbiny et al. [61]. In commercial liquid feed enzymes not mixed into a diet, after 7.5 months of storage, cellulase activity was 100 and 75% of their original value when stored at 4°C and 30 °C, respectively, while xylanase residual activity was reduced to 20% and 50% at 30 °C and 4 °C, respectively.

Cellulase activity produced by *Paenibacillus chitinolyticus* decreased by 33% after 9 days of storage at 4 °C [62]. The storage of cellulase from *Aspergillus terreus* at 5 °C kept 75% of its initial activity after 39 weeks of storage, while stored at RT, it only retained 40% of the initial activity [63]. In this work, cellulase activity was reduced by 50% after 19 weeks at RT and after 31 weeks at 4 °C.

In the present study, xylanase was more stable at 4 °C than RT. However, the *t*_1/2_ was only delayed by 9 days. Previously, the shelf-life of xylanase produced by pseudomonas sp. XPB-6 was studied at RT, and at 4 °C. Xylanase maintained its activity for 25 days at 4°C, which was reduced to 16 days if stored at 25 °C, gradually decreasing its activity afterward [64]. According to Yadav et al. [65], xylanase activity from *Anoxybacillus kamchatkensis* NASTPD13 remained stable for 25 days at 4 °C, decreasing 70% of its initial activity after 6 weeks. This enzyme was also stable for 15 days at RT, and its activity decreased by 40% after 6 weeks. An extracellular and purified cellulase-free xylanase produced by alkalophilic *Bacillus subtilis* ASH kept its activity for 6 weeks when stored at 4 °C, decreasing afterward. Nonetheless, the enzyme retained 80% of the initial activity after 10 weeks of storage at 4 °C [66]. In this study, xylanase activity was reduced by 50% after 10 weeks of storage at 4 °C and 9 weeks at RT.

Protease activity is one of the factors responsible for the deactivation of xylanases and cellulases [61]. In this study, the protease activity was evaluated but not detected in the diets. Thus, the temperature was the main factor in the deactivation of enzymes during storage.

### 3.2. Antioxidant Activity

**Figure 3:**
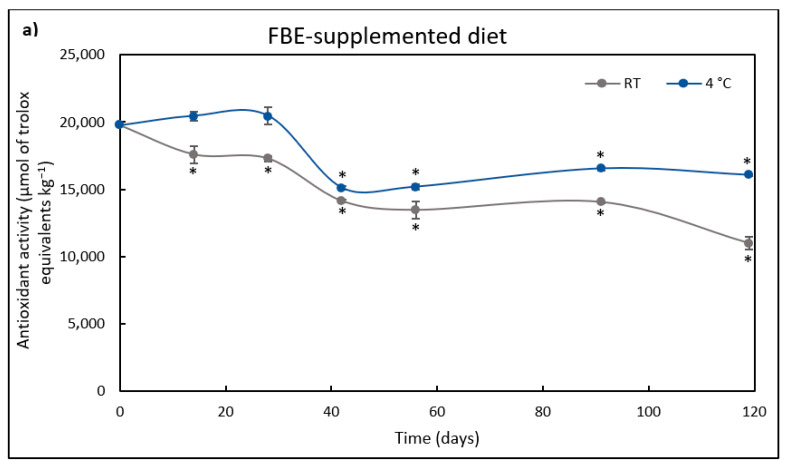

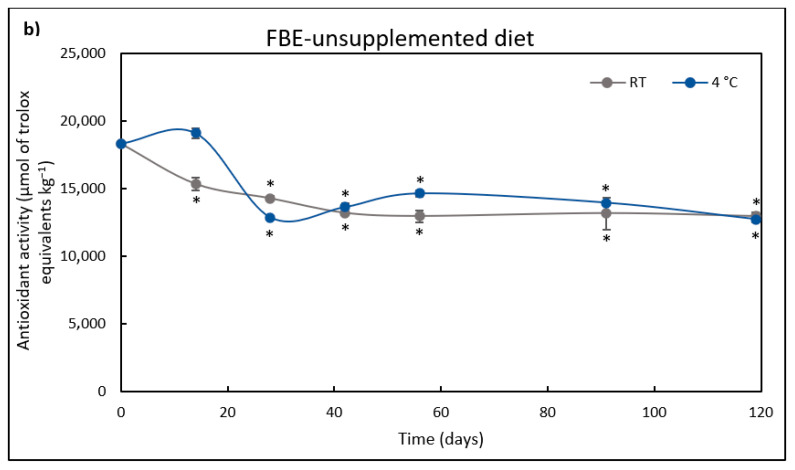
Antioxidant activity (µmol/kg) of FBE-supplemented diet (**a**) over storage time (days) at room temperature (RT) and 4 °C. The results represent the average of three independent. Antioxidant activity (µmol/kg) of FBE-unsupplemented diet (**b**) over storage time (days) at room temperature (RT) and 4 °C. The results represent the average of three independent experiments and the error bars represent SD. The asterisk denotes a significant difference (*p* < 0.05) from the initial antioxidant activity.

The antioxidant activity of FBE extract-unsupplemented (control) or supplemented diets during storage at RT and 4 °C is presented in Figure 3.

Even though not supplemented with the FBE, the control diet had some antioxidant activity, reaching 18357 μmol TE kg^−1^ of diet. This antioxidant activity can be attributed to the antioxidant potential of the feed ingredients as plant-based ingredients, namely sunflower, soybean, and rapeseed meal. Indeed, Sousa et al. [67] evaluated the antioxidant activity of these plant feedstuffs and observed that sunflower meal had the higher antioxidant activity (73 μmol TE g^−1^), followed by rapeseed meal (36 μmol TE g^−1^) and soybean meal (12 μmol TE g^−1^).

At time 0 h, the FBE-supplemented diet had 7.5% more antioxidant activity than the control diet and maintained its antioxidant activity without any significant difference for 42 days when stored at 4 °C. The antioxidant activity of the control diet decreased after 14 days at RT, and after 28 days at 4 °C. Afterward, the control diet maintained the antioxidant activity until day 119 of storage. On the other hand, in the FBE-supplemented diet stored at 4 °C, the antioxidant activity decreased after the 42nd day, remaining stable after this period. When stored at RT, the FBE-supplemented diet antioxidant activity significantly decreased compared to the initial time (0 h) after the 14th day.

After 119 days, the FBE-supplemented diet antioxidant activity was 1.5-fold higher when stored at 4 °C than at RT. Moreover, even after 119 days of storage, the antioxidant activity of the FBE-supplemented diet was 1.3-fold higher than that of the control diet stored at RT or 4 °C. The control diet showed a similar decrease in antioxidant activity after 119 days at both temperatures (30% relative to the initial activity). However, the FBE-supplemented diet lost 44% and 18% of the initial antioxidant activity when stored at RT or 4 °C, respectively. This fluctuation in activity may be due to the degradation of antioxidant compounds due to light and heat. Thus, storing the FBE-supplemented diet at 4 °C is beneficial for preserving antioxidant activity.

The stability of polyphenols under different storage and processing conditions is crucial and should be considered to ensure that these compounds possess the desired properties and maintain their activity under different conditions, which can involve high temperatures and light [68]. This is particularly important during long-term diet storage, as antioxidants are used to protect against lipid oxidation, especially if stored at elevated temperatures. High temperatures and light can also affect other sources of antioxidant activity, such as vitamins and those present in plant feedstuffs [69]. So, the adequate dietary antioxidant level should account for its consumption during storage and transport periods [33].

Multiple storage factors, such as light exposure, temperature, oxygen, and pH, have been highlighted to compromise the stability of the phenolic compounds [70,71]. The storage temperature is one of the major factors affecting the stability of antioxidant activity in diets. For example, an aqueous extract from *Piper betle* kept 96% of its antioxidant activity after 180 days of storage at 5 °C; after 75 days at 25 °C, the activity was reduced to 90% [72]. In this case, the high retention of antioxidant activity in the extract was due to the inclusion of maltodextrin as a carrier agent. Galmarini et al. [73] studied the thermostability of several red wine powder phenolic compounds for 70 days of storage at 28 °C and 38 °C and found that thermostability depended on the phenolic compound. Malvidin 3-G, epicatechin gallate, catechin, and epicatechin mainly decreased at the highest temperature; epigallocatechin decreased at both temperatures, while caffeic acid, gallic acid, caftaric acid, quercetin 3-G, and resveratrol were not reduced in the studied conditions.

### 3.3. Inhibition of Lipid Peroxidation

The composition of diets affects their susceptibility to oxidation. In the present study, experimental diets are composed of 12.3% fish oil (Table 1), which is particularly prone to oxidation given its high content of unsaturated fatty acids. Feeding oxidized lipids to fish can cause severe issues such as damage to the intestinal tract and other organs, as well as muscular dystrophy, among others [74]. These effects will be reflected in the organisms’ performance, decreasing feed consumption, reducing animal weight, feed conversion ratio, and increased fish disease susceptibility and mortality [33]. The inhibition of lipid peroxidation through dietary antioxidant supplementation is an essential factor in preserving fish feed quality. Moreover, dietary supplementation with antioxidants has been demonstrated as a novel approach to maintaining cell metabolism and neutralizing excess free radicals in fish under oxidative stress conditions [75].

**Figure 4:**
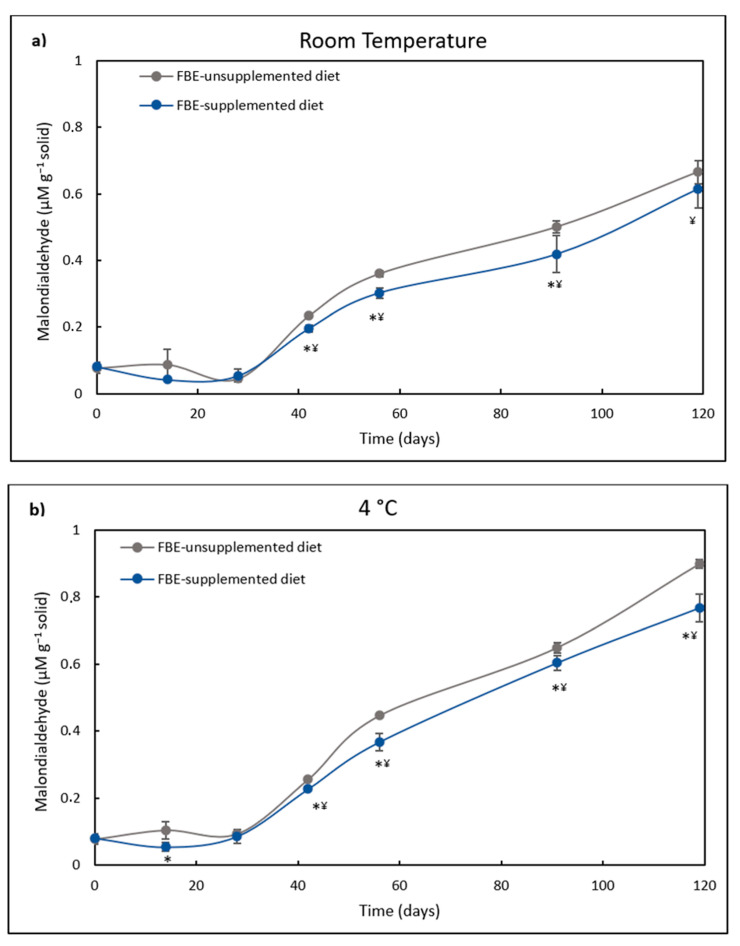
(**a**). Lipid peroxidation (µM g^−1^ solid) of FBE-supplemented diet and FBE-unsupplemented diet (control diet) over storage time (days) at room temperature (RT). The results represent the average of three independent experiments and the error bars represent SD. The asterisk denotes a significant difference (*p* < 0.05) between both diets at each time. ¥ denotes a significant difference (*p* < 0.05) from the initial lipid peroxidation level. (**b**). Lipid peroxidation (µM g^−1^ solid) of FBE-supplemented diet and FBE-unsupplemented diet (control diet) over storage time (days) at 4 °C. The results represent the average of three independent experiments and the error bars represent SD. The asterisk denotes a significant difference (*p* < 0.05) between both diets at each time. ¥ denotes a significant difference (*p* < 0.05) from the initial lipid peroxidation level.

In the present study, after 28 days of storage, lipid peroxidation of FBE-unsupplemented and supplemented diets maintained a similar level (Figure 4). However, in diets kept at 4 °C, after 42 days, lipid peroxidation of the FBE-supplemented diet lead was lower than the control diet. In diets stored at RT, the lipid peroxidation of the FBE-supplemented diet was lower than that of the control diet from the 41st to the 91st day of storage. The highest difference in lipid peroxidation between both diets was observed after 119 days of storage at 4 °C, where the lipid peroxidation of the control diet was 17% higher than that of the FBE-supplemented diet. This result correlates well with the higher antioxidant activity of the FBE diet over the control after 119 days of storage.

Both the addition of antioxidant additives and storage temperature affect the lipid oxidation of fisheries products. The effect of freeze-dried red grape pomace addition to minced frozen horse mackerel stored at −20 °C on lipid peroxidation was studied [76]. The authors observed that this additive delayed lipid oxidation of minced horse mackerel muscle during the first 3 months of frozen storage. The control (without supplementation) significantly increased lipid oxidation throughout the storage time. In another study, the levels of TBA of fishmeal were evaluated during its storage at RT and 4 °C [77]. After 135 days of storage, fish meal TBA levels increased to 16 and 10 mg kg^−1^ at RT and 4 °C, respectively.

## 4. Conclusions

The results of this study allowed to evaluate the behavior of bioactive compounds added to diets for aquaculture during storage. During storage, cellulase activity was more stable than xylanase. Storage temperature, room temperature or 4 °C, significantly affected the deactivation rate of enzymes and at the end of the trial, these enzymes were reduced up to 79% and 42% for xylanase and cellulase, respectively, in both temperatures.

The antioxidant activity of the FBE-supplemented diet was more stable for an extended period than that of the control diet. The FBE-supplemented diet stored at 4 °C kept the higher antioxidant activity even after 119 days of storage. The FBE helped reduce lipid peroxidation in the later stages of storage.

## Figures and Tables

**Table 1:** Ingredient and proximate analyses of experimental diets.

	FBE-Unsupplemented Diet	FBE-Supplemented Diet
Ingredients (% dry matter)		
Fish meal	9.8	9.8
Soluble fish protein concentrate	4.9	4.9
Wheat gluten meal	18.6	18.6
Soybean meal	9.8	9.8
Rice bran meal	9.8	9.8
Sunflower meal	8.8	8.8
Rapeseed meal	7.8	7.8
Wheat meal	6.3	6.1
Hemoglobin	4.9	4.9
Taurine	0.5	0.5
Lysine	0.6	0.6
Methionine	0.2	0.2
Fish oil	12.3	12.3
Vitamin premix ^1^	1.0	1.0
Choline chloride (50%)	0.5	0.5
Mineral premix ^2^	1.0	1.0
Binder ^3^	1.0	1.0
CaHPO ^4^	1.0	1.0
Hydrolyzed shrimp	1.2	1.2
FBE ^4^		0.26
Proximate analyses (% dry weight)		
Dry matter (%)	92.9	92.3
Crude protein	47.9	48.1
Crude lipids	16.3	16.4
Starch	2.56	2.58
Ash	6.8	6.7
Gross energy (kJ g^−1^)	24.1	24.2
Antioxidant activity (µmol of Trolox equivalents g^−1^)	18.4	19.8
Enzymatic activity (U g^−1^)		
Xylanase	0	2.61
Cellulase	0	1.08

^1^ Vitamins premix (mg kg^−1^ diet): retinol. 18.000 (IU kg^−1^ diet); calciferol. 2000 (IU kg^−1^ diet); alpha-tocopherol. 35; menadione sodium bis. 10; thiamin. 15; riboflavin. 25; Ca pantothenate. 50; nicotinic acid. 200; pyridoxine. 5; folic acid. 10; cyanocobalamin. 0.02; biotin. 1.5; ascorbyl monophosphate. 50; inositol. 400. ^2^ Minerals (mg kg^−1^ diet): cobalt sulfate. 1.91; copper sulfate. 19.6; iron sulfate. 200; sodium fluoride. 2.21; potassium iodide. 0.78; magnesium oxide. 830; manganese oxide. 26; sodium selenite. 0.66; zinc oxide. 37.5; dibasic calcium phosphate. 5.9 (g kg^−1^ diet); potassium chloride. 1.15 (g kg^−1^ diet); sodium chloride. 0.4 (g kg^−1^ diet). ^3^ Binder (Aquacube. Agil. England). ^4^ FBE: Lyophilized extract produced by solid-state fermentation of the olive mill and winery by-products by *Aspergillus ibericus* MUM 03.49.

**Table 2:** Inactivation kinetic parameters of cellulase and xylanase stored at room temperature and 4 °C.

	*K_d_* (Days^−1^)		*t*_1/2_ (Days)		D-Value (Days)		R^2^	
Enzyme	RT	4 °C	RT	4 °C	RT	4 °C	RT	4 °C
Cellulase	0.0051	0.032	136	217	451	720	0.8963	0.8133
Xylanase	0.011	0.0096	63	72	209	240	0.9257	0.866

*Kd*: Thermal inactivation rate constant; *t*_1/2_: half-life; D-Value: Decimal reduction time; R^2^: coefficient of determination of first-order model.

## Data Availability

The raw data supporting the conclusions of this article will be made available by the authors without undue reservation.

## References

[1] Hua K., Cobcroft J.M., Cole A., Condon K., Jerry D.R., Mangott A., Praeger C., Vucko M.J., Zeng C., Zenger K. (2019). The Future of Aquatic Protein: Implications for Protein Sources in Aquaculture Diets. One Earth.

[2] Sofia (2018). Seafish Insight: Fishmeal Production and Trends.

[3] Glencross B., Fracalossi D., Hua K., Izquierdo M., Ma K., Overland M., Robb D., Roubach R., Schrama J., Small B. Harvesting the Benefits of Nutritional Research to Address Global Challenges in the 21st Century 2020. Proceedings of the Global Conference on Aquaculture 2020.

[4] Francis G., Makkar H.P.S., Becker K. (2001). Antinutritional Factors Present in Plant-Derived Alternate Fish Feed Ingredients and Their Effects in Fish. Aquaculture.

[5] Sinha A.K., Kumar V., Makkar H.P.S., de Boeck G., Becker K. (2011). Non-Starch Polysaccharides and Their Role in Fish Nutrition—A Review. Food Chem..

[6] Castillo S., Gatlin D.M. (2015). Dietary Supplementation of Exogenous Carbohydrase Enzymes in Fish Nutrition: A Review. Aquaculture.

[7] Encarnação P., Nates S.F. (2016). 5-Functional Feed Additives in Aquaculture Feeds. Aquafeed Formulation.

[8] Fernandes H., Salgado J.M., Martins N., Peres H., Oliva-Teles A., Belo I. (2019). Sequential Bioprocessing of Ulva Rigida to Produce Lignocellulolytic Enzymes and to Improve Its Nutritional Value as Aquaculture Feed. Bioresour. Technol..

[9] Kolkovski S., Czesny S., Dabrowski K. (2000). Use of Krill Hydrolysate as a Feed Attractant for Fish Larvae and Juveniles. J. World Aquac. Soc..

[10] Gatlin D.M., Barrows F.T., Brown P., Dabrowski K., Gaylord T.G., Hardy R.W., Herman E., Hu G., Krogdahl Å., Nelson R. (2007). Expanding the Utilization of Sustainable Plant Products in Aquafeeds: A Review. Aquac. Res..

[11] Oliva-Teles A., Gonçalves P. (2001). Partial Replacement of Fishmeal by Brewers Yeast (*Saccaromyces cerevisae*) in Diets for Sea Bass (*Dicentrarchus labrax*) Juveniles. Aquaculture.

[12] Hardy R.W. (2010). Utilization of Plant Proteins in Fish Diets: Effects of Global Demand and Supplies of Fishmeal. Aquac. Res..

[13] Gatesoupe F.J. (1999). The Use of Probiotics in Aquaculture. Aquaculture.

[14] Ringø E., Olsen R.E., Gifstad T.Ø., Dalmo R.A., Amlund H., Hemre G.-I., Bakke A.M. (2010). Prebiotics in Aquaculture: A Review. Aquac. Nutr..

[15] Dawood M.A.O., Koshio S., Esteban M.Á. (2018). Beneficial Roles of Feed Additives as Immunostimulants in Aquaculture: A Review. Rev. Aquac..

[16] Citarasu T. (2010). Herbal Biomedicines: A New Opportunity for Aquaculture Industry. Aquac. Int..

[17] Hassan A.M., Kenawy A.M., Abbas W.T., Abdel-Wahhab M.A. (2010). Prevention of Cytogenetic, Histochemical and Biochemical Alterations in Oreochromis Niloticus by Dietary Supplement of Sorbent Materials. Ecotoxicol. Environ. Saf..

[18] Volpatti D., Chiara B., Francesca T., Marco G. (2013). Growth Parameters, Innate Immune Response and Resistance to Listonella (Vibrio) Anguillarum of Dicentrarchus Labrax Fed Carvacrol Supplemented Diets. Aquac. Res..

[19] Bampidis V., Azimonti G., Bastos M.d.L., Christensen H., Dusemund B., Kouba M., Kos Durjava M., López-Alonso M., López Puente S., Marcon F. (2019). Safety and Efficacy of HOSTAZYM^®^ X (Endo-1,4-beta-xylanase) as a Feed Additive for Rabbits for Fattening. EFSA J..

[20] Silano V., Barat Baviera J.M., Bolognesi C., Cocconcelli P.S., Crebelli R., Gott D.M., Grob K., Lampi E., Mortensen A., Riviere G. (2019). Safety Evaluation of the Food Enzyme Cellulase from Trichoderma Reesei (Strain DP-Nzc36). EFSA J..

[21] Guan D., Wang Z., Han H., Sun H., Li Y., Wan W., Wang J. (2021). Effects of Nonstarch Polysaccharide Hydrolase of Plant Protein-Based Diets on Growth, Nutrient Digestibility, and Protease/Amylase Activities of Yellow River Carp, Cyprinus Carpio. J. World Aquac. Soc..

[22] Gericke S.J., Salie K., de Wet L., Goosen N.J. (2021). Effects of Dietary Supplementation of Endo-(1,4)-β-Xylanase in Plant-Based Diets on Growth Performance, Hindgut Microbial Diversity, and Blood Chemistry in Large on-Growing African Catfish (*Clarias gariepinus*). J. Appl. Aquac..

[23] Maas R.M., Verdegem M.C.J., Stevens T.L., Schrama J.W. (2020). Effect of Exogenous Enzymes (Phytase and Xylanase) Supplementation on Nutrient Digestibility and Growth Performance of Nile Tilapia (*Oreochromis niloticus*) Fed Different Quality Diets. Aquaculture.

[24] Magalhães R., Coutinho F., Pousão-Ferreira P., Aires T., Oliva-Teles A., Peres H. (2015). Corn Distiller’s Dried Grains with Solubles: Apparent Digestibility and Digestive Enzymes Activities in European Seabass (*Dicentrarchus labrax*) and Meagre (*Argyrosomus regius*). Aquaculture.

[25] Magalhães R., Lopes T., Martins N., Díaz-Rosales P., Couto A., Pousão-Ferreira P., Oliva-Teles A., Peres H. (2016). Carbohydrases Supplementation Increased Nutrient Utilization in White Seabream (*Diplodus sargus*) Juveniles Fed High Soybean Meal Diets. Aquaculture.

[26] Diógenes A.F., Castro C., Carvalho M., Magalhães R., Estevão-Rodrigues T.T., Serra C.R., Oliva-Teles A., Peres H. (2018). Exogenous Enzymes Supplementation Enhances Diet Digestibility and Digestive Function and Affects Intestinal Microbiota of Turbot (*Scophthalmus maximus*) Juveniles Fed Distillers’ Dried Grains with Solubles (DDGS) Based Diets. Aquaculture.

[27] Amerah A.M., Gilbert C., Simmins P.H., Ravindran V. (2019). Influence of Feed Processing on the Efficacy of Exogenous Enzymes in Broiler Diets. World’s Poult. Sci. J..

[28] Almaraz-Sánchez I., Amaro-Reyes A., Acosta-Gallegos J.A., Mendoza-Sánchez M. (2022). Processing Agroindustry By-Products for Obtaining Value-Added Products and Reducing Environmental Impact. J. Chem..

[29] Zhuang J., Marchant M.A., Nokes S.E., Strobel H.J. (2007). Economic Analysis of Cellulase Production Methods for Bio-Ethanol. Appl. Eng. Agric..

[30] Thomas L., Larroche C., Pandey A. (2013). Current Developments in Solid-State Fermentation. Biochem. Eng. J..

[31] Pandey A. (2003). Solid-State Fermentation. Biochem. Eng. J..

[32] Leite P., Sousa D., Fernandes H., Ferreira M., Costa A.R., Filipe D., Gonçalves M., Peres H., Belo I., Salgado J.M. (2020). Recent Advances in Production of Lignocellulolytic Enzymes by Solid-State Fermentation of Agro-Industrial Wastes. Curr. Opin. Green Sustain. Chem..

[33] Andrews J.T., Giesen A.F., Scott F.R. (2004). Antioxidants Manage Effects of Oxidation on Feeds, Feed Ingredients. Glob. Aquac. Advocate.

[34] Choe E., Min D.B. (2009). Mechanisms of Antioxidants in the Oxidation of Foods. Compr. Rev. Food Sci. Food Saf..

[35] Godwin A., Ramachandra Prabhu H. (2006). Lipid Peroxidation of Fish Oils. Indian J. Clin. Biochem..

[36] Sargent J.R., Tocher D.R., Bell J.G. (2002). The Lipids. Fish Nutrition.

[37] Mourente G., Bell J.G. (2006). Partial Replacement of Dietary Fish Oil with Blends of Vegetable Oils (Rapeseed, Linseed and Palm Oils) in Diets for European Sea Bass (*Dicentrarchus labrax* L.) over a Long Term Growth Study: Effects on Muscle and Liver Fatty Acid Composition and Effectiveness of a Fish Oil Finishing Diet. Comp. Biochem. Physiol.-B Biochem. Mol. Biol..

[38] Hossen M., Das M., Sumi K., Hasan M. (2013). Effect of Storage Time on Fish Feed Stored at Room Temperature and Low Temperature. Progress. Agric..

[39] Hernández A., García García B., Jordán M.J., Hernández M.D. (2014). Natural Antioxidants in Extruded Fish Feed: Protection at Different Storage Temperatures. Anim. Feed Sci. Technol..

[40] Hamre K., Kolås K., Sandnes K. (2010). Protection of Fish Feed, Made Directly from Marine Raw Materials, with Natural Antioxidants. Food Chem..

[41] Lundebyea A.K., Hovea H., Mågea A., Bohneb V.J.B., Hamrea K. (2010). Levels of Synthetic Antioxidants (Ethoxyquin, Butylated Hydroxytoluene and Butylated Hydroxyanisole) in Fish Feed and Commercially Farmed Fish. Food Addit. Contam. Part A Chem. Anal. Control. Expo. Risk Assess..

[42] Bampidis V., Azimonti G., Bastos M.d.L., Christensen H., Dusemund B., Fašmon Durjava M., Kouba M., López-Alonso M., López Puente S., Marcon F. (2022). Safety and Efficacy of a Feed Additive Consisting of Ethoxyquin (6-ethoxy-1,2-dihydro-2,2,4-trimethylquinoline) for All Animal Species (FEFANA Asbl). EFSA J..

[43] EUR-Lex-32021R0412-EN-EUR-Lex. https://eur-lex.europa.eu/eli/reg_impl/2021/412/oj.

[44] Olive Oil. https://agriculture.ec.europa.eu/farming/crop-productions-and-plant-based-products/olive-oil_en.

[45] Wine. https://agriculture.ec.europa.eu/farming/crop-productions-and-plant-based-products/wine_en.

[46] Devesa-Rey R., Vecino X., Varela-Alende J.L., Barral M.T., Cruz J.M., Moldes A.B. (2011). Valorization of Winery Waste vs. the Costs of Not Recycling. Waste Manag..

[47] Tapia-Quirós P., Montenegro-Landívar M.F., Reig M., Vecino X., Alvarino T., Cortina J.L., Saurina J., Granados M. (2020). Olive Mill and Winery Wastes as Viable Sources of Bioactive Compounds: A Study on Polyphenols Recovery. Antioxidants.

[48] Wallace G., Fry S.C. (1994). Phenolic Components of the Plant Cell Wall. Int. Rev. Cytol..

[49] Martins S., Mussatto S.I., Martínez-Avila G., Montañez-Saenz J., Aguilar C.N., Teixeira J.A. (2011). Bioactive Phenolic Compounds: Production and Extraction by Solid-State Fermentation. A Review. Biotechnol. Adv..

[50] Verduzco-Oliva R., Gutierrez-Uribe J.A. (2020). Beyond Enzyme Production: Solid State Fermentation (SSF) as an Alternative Approach to Produce Antioxidant Polysaccharides. Sustainability.

[51] Yang W., Yang Y., Zhang L., Xu H., Guo X., Yang X., Dong B., Cao Y. (2017). Improved Thermostability of an Acidic Xylanase from Aspergillus Sulphureus by Combined Disulphide Bridge Introduction and Proline Residue Substitution. Sci. Rep..

[52] Moura G.d.S., Teixeira Lanna E.A., Donzele J.L., Falkoski D.L., de Rezende S.T., Oliveira M.G.d.A., Albino L.F.T. (2016). Stability of Enzyme Complex Solid-State Fermentation Subjected to the Processing of Pelleted Diet and Storage Time at Different Temperatures. Rev. Bras. Zootec..

[53] Filipe D., Fernandes H., Castro C., Peres H., Oliva-Teles A., Belo I., Salgado J.M. (2019). Improved Lignocellulolytic Enzyme Production and Antioxidant Extraction Using Solid-state Fermentation of Olive Pomace Mixed with Winery Waste. Biofuels Bioprod. Biorefining.

[54] Miller G.L. (1959). Modified DNS Method for Reducing Sugars. Anal. Chem..

[55] Dulf F.V., Vodnar D.C., Dulf E.-H., Toşa M.I. (2015). Total Phenolic Contents, Antioxidant Activities, and Lipid Fractions from Berry Pomaces Obtained by Solid-State Fermentation of Two Sambucus Species with Aspergillus Niger. J. Agric. Food Chem..

[56] Nguyen T.T.K., Laosinwattana C., Teerarak M., Pilasombut K. (2017). Potential Antioxidant and Lipid Peroxidation Inhibition of Phyllanthus Acidus Leaf Extract in Minced Pork. Asian-Australas J. Anim. Sci..

[57] Pal A., Khanum F. (2010). Characterizing and Improving the Thermostability of Purified Xylanase from Aspergillus Niger DFR-5 Grown on Solid-State-Medium. J. Biochem. Technol..

[58] Fernandes H., Salgado J.M., Ferreira M., Vršanská M., Fernandes N., Castro C., Oliva-Teles A., Peres H., Belo I. (2022). Valorization of Brewer’s Spent Grain Using Biological Treatments and Its Application in Feeds for European Seabass (*Dicentrarchus labrax*). Front. Bioeng. Biotechnol..

[59] Adeola O., Cowieson A.J. (2011). BOARD-INVITED REVIEW: Opportunities and Challenges in Using Exogenous Enzymes to Improve Nonruminant Animal Production. J. Anim. Sci..

[60] Fuente J.M., Perez De Ayala P., Flores A., Villamide M.J. (1998). Effect of Storage Time and Dietary Enzyme on the Metabolizable Energy and Digesta Viscosity of Barley-Based Diets for Poultry. Poult. Sci..

[61] El-Sherbiny M., El-Chaghaby G. (2012). Storage Temperature and Stabilizers in Relation to the Activity of Commercial Liquid Feed Enzymes: A Case Study from Egypt. J. Agrobiol..

[62] Buntić A.V., Pavlović M.D., Antonović D.G., Šiler-Marinković S.S., Dimitrijević-Branković S.I. (2016). Utilization of Spent Coffee Grounds for Isolation and Stabilization of Paenibacillus Chitinolyticus CKS1 Cellulase by Immobilization. Heliyon.

[63] Maragathavalli S., Megha S., Brindha S., Karthikeyan V.V., Annadurai S.G. (2015). Effect of Different Nutritive Sources for Enhancing Cellulose Producion in Aspergillus Terreus. Int. J. Sci. Nat..

[64] Sharma P.K., Chand D. (2012). Production of Cellulase Free Thermostable Xylanase from Pseudomonas sp. XPB-6. Int. Res. J. Biol. Sci..

[65] Yadav P., Maharjan J., Korpole S., Prasad G.S., Sahni G., Bhattarai T., Sreerama L. (2018). Production, Purification, and Characterization of Thermostable Alkaline Xylanase from Anoxybacillus Kamchatkensis NASTPD13. Front. Bioeng. Biotechnol..

[66] Sanghi A., Garg N., Gupta V.K., Mittal A., Kuhad R.C. (2010). One-Step Purification and Characterization of Cellulase-Free Xylanase Produced by Alkalophilic Bacillus Subtilis Ash. Braz. J. Microbiol..

[67] Sousa D., Salgado J.M., Cambra-López M., Dias A.C.P., Belo I. (2022). Degradation of Lignocellulosic Matrix of Oilseed Cakes by Solid-State Fermentation: Fungi Screening for Enzymes Production and Antioxidants Release. J. Sci. Food Agric..

[68] Volf I., Ignat I., Neamtu M., Popa V. (2014). Thermal Stability, Antioxidant Activity, and Photo-Oxidation of Natural Polyphenols. Chem. Pap..

[69] Yang P., Wang H.K., Zhu M., Li L.X., Ma Y.X. (2021). Degradation Kinetics of Vitamins in Premixes for Pig: Effects of Choline, High Concentrations of Copper and Zinc, and Storage Time. Anim. Biosci..

[70] Sharma R.J., Gupta R.C., Singh S., Bansal A.K., Singh I.P. (2016). Stability of Anthocyanins- and Anthocyanidins-Enriched Extracts, and Formulations of Fruit Pulp of Eugenia Jambolana (‘Jamun’). Food Chem..

[71] Kearsley M.W., Rodriguez N. (2007). The Stability and Use of Natural Colours in Foods: Anthocyanin, β-Carotene and Riboflavin. Int J. Food Sci. Technol..

[72] Ali A., Chong C.H., Mah S.H., Abdullah L.C., Choong T.S.Y., Chua B.L. (2018). Impact of Storage Conditions on the Stability of Predominant Phenolic Constituents and Antioxidant Activity of Dried Piper Betle Extracts. Molecules.

[73] Galmarini M.V., Maury C., Mehinagic E., Sanchez V., Baeza R.I., Mignot S., Zamora M.C., Chirife J. (2013). Stability of Individual Phenolic Compounds and Antioxidant Activity During Storage of a Red Wine Powder. Food Bioprocess Technol..

[74] Bacou E., Walk C., Rider S., Litta G., Perez-Calvo E. (2021). Dietary Oxidative Distress: A Review of Nutritional Challenges as Models for Poultry, Swine and Fish. Antioxidants.

[75] Sechi S., Fiore F., Chiavolelli F., Dimauro C., Nudda A., Cocco R. (2017). Oxidative Stress and Food Supplementation with Antioxidants in Therapy Dogs. Can. J. Vet. Res..

[76] Sánchez-Alonso I., Jiménez-Escrig A., Saura-Calixto F., Borderías A.J. (2007). Effect of Grape Antioxidant Dietary Fibre on the Prevention of Lipid Oxidation in Minced Fish: Evaluation by Different Methodologies. Food Chem..

[77] Lin H., Yang Q., Wang A., Wang J., Tan B., Ray G.W., Dong X., Chi S., Liu H., Zhang S. (2021). Effects of Fish Meal under Different Storage Conditions on Growth, Serum Biochemical Indices and Antioxidant Capacity for Juvenile Grouper Epinephelus Coioides. Aquac. Nutr..

